# Predictors of Salivary Fistulas in Patients Undergoing Salvage Total Laryngectomy

**DOI:** 10.1155/2014/373825

**Published:** 2014-11-20

**Authors:** Shethal Bearelly, Steven J. Wang

**Affiliations:** Department of Otolaryngology-Head and Neck Surgery, University of California, San Francisco, 2333 Post Street, 3rd Floor, San Francisco, CA 94143, USA

## Abstract

*Background*. Salivary fistula is a common complication after salvage total laryngectomy. Previous studies have not considered the number of layers of pharyngeal closure and have not classified fistulas according to severity. Our objective was to analyze our institutional experience with salvage total laryngectomy, categorize salivary fistulas based on severity, and study the effect of various pharyngeal closure techniques on fistula incidence. *Methods*. Retrospective analysis of 48 patients who underwent salvage total laryngectomy, comparing pharyngeal closure technique and use of a pectoralis major flap with regard to salivary fistula rate. Fistulas were categorized into major and minor fistulas based on whether operative intervention was required. *Results*. The major fistula rate was 18.8% (9/48) and the minor fistula rate was 29.2% (14/48). The overall (major plus minor) fistula rate was 47.9%. The overall fistula and major fistula rates decreased with increasing the number of closure layers and with use of a pectoralis major flap; however, these correlations did not reach statistical significance. Other than age, there were no clinicopathologic variables associated with salivary fistulas. *Conclusion*. For salvage total laryngectomies, increasing the number of closure layers or use of a pectoralis major flap may reduce the risk of salivary fistula.

## 1. Introduction

The incidence of laryngeal cancer has decreased in the USA in recent years as rates of smoking have declined. However, larynx cancer continues to be a serious problem for individuals suffering from this disease, with treatment frequently affecting the patient's ability to phonate and swallow. This year, 12630 people in the USA are estimated to be diagnosed with laryngeal cancer and 3610 will die from this disease [1].

The Veterans Affairs laryngeal cancer study [2] and the Radiation Therapy Oncology Group trial 91-11 [3] are the basis for the organ-preservation treatment approaches currently employed for advanced laryngeal cancer. Definitive radiation treatment with chemotherapy is utilized as the initial treatment strategy for many advanced laryngeal cancers except those with cartilage involvement. However, when there is persistence of disease or recurrence of cancer after chemoradiation, salvage total laryngectomy is often necessary to achieve cure.

Pharyngocutaneous or salivary fistula is a common complication after salvage total laryngectomy and can lead to serious consequences. Fistulas can lead to infection and skin breakdown, prolonging the patient's hospital stay, at times necessitating operative repair. In very serious cases, the persistent bathing of saliva around major vessels can lead to arterial erosion and subsequent carotid blowout.

The reported incidence of pharyngocutaneous fistula for primary total laryngectomy varies from 10 to 35% [4–13]. For salvage total laryngectomy, the reported fistula rate is generally higher, varying in the literature from 25 to 50% [14–19]. Many groups have attempted to identify the risk factors for salivary fistula with salvage total laryngectomy, and associations have been reported for poor preoperative nutrition, low hemoglobin, prior tracheostomy, liver disease, and diabetes [4, 12, 16]. With some exceptions, most groups have found that a prior history of radiation and/or chemotherapy predisposes patients to a higher risk of salivary fistula after total laryngectomy [9–11, 20–22]. In an attempt to reduce the incidence of salivary fistula after salvage total laryngectomy, the use of pedicled or free vascularized tissue transfer to reinforce the neopharyngeal closure has been studied. While there is some data that vascularized flaps may provide benefit, the conclusions have thus far been conflicting [6, 23–26].

This study aims to identify what factors play a role in development of pharyngocutaneous or salivary fistulas in patients undergoing salvage total laryngectomy. A secondary aim was to separately analyze the predictive factors for development of minor fistulas that are managed conservatively and major fistulas that are severe enough to require surgical intervention.

## 2. Methods

A retrospective chart review was performed for all patients who underwent salvage total laryngectomy for laryngeal squamous cell carcinoma at the University of California, San Francisco (UCSF). The Committee on Human Research at UCSF granted approval for this study.

We included all patients who had laryngeal squamous cell carcinoma treated primarily with radiation therapy or chemoradiation who subsequently were found to have recurrent or persistent disease, requiring a salvage total laryngectomy between January 1, 2002, and January 1, 2012. Oncologic resection was completed within the Department of Otolaryngology-Head and Neck Surgery at UCSF. During this time period, eight different attending surgeons performed these laryngectomies.

Data collection was performed using all electronic medical record systems in place at UCSF, and patient records were screened for inclusion in this study using procedure codes for total laryngectomy. These records were then reviewed to include only those who underwent salvage total laryngectomy. Patients who had salvage total laryngectomy for reasons other than cancer such as chronic aspiration or dysfunctional larynx were excluded. Demographic data was then collected including information about sex, race, ethnicity, and tobacco or alcohol use. Oncologic data regarding the primary tumor including the American Joint Committee on Cancer (AJCC) stage, TNM stage, and detailed histopathologic data were collected. The use of adjuvant chemotherapy was also collected.

Salvage laryngectomy surgical details regarding extent of pharyngeal resection or concurrent neck dissection were reviewed. The type of neopharyngeal closure was also evaluated with regard to whether a single-, double-, or triple-layer closure was performed. Data regarding use of pedicled pectoralis muscle flaps or free tissue transfer was also collected. It was noted whether an onlay or inlay pectoralis flap was used. Inlay method refers to using the skin paddle of the pectoralis myocutaneous flap to reconstruct a portion of the neopharyngeal wall. The onlay method refers to placing a pectoralis myofascial flap on top of the neopharyngeal closure without actually augmenting the neopharynx wall.

The occurrence of a salivary fistula for each of these patients was determined by reviewing the discharge summary for the hospital stay following the salvage total laryngectomy as well as the clinic note for the first postoperative visit. A fistula was defined as any documented clinical suspicion or clear evidence of salivary leak, on a continuum from erythema of the neck to saliva within the surgical drain to frank wound breakdown and leakage of saliva. Salivary fistulas were further categorized into major salivary fistula and minor salivary fistula. Major salivary fistulas were defined as those that needed revision surgery for closure of the leak. Minor salivary fistulas were defined as those that resolved with nonoperative management.

Data analyses were performed with SAS, Version 9.3 (SAS Institute, Cary, North Carolina). All categorical data were analyzed by using Chi-square and Fisher exact tests. Normally distributed data were analyzed using independent sample *t*-testing and nonparametric data using Mann-Whitney testing. Statistical significance was defined as *P* value <0.05.

## 3. Results

During the study period, there were 181 patients who underwent a total laryngectomy. Of these, 133 patients were excluded because they did not receive prior radiation or chemoradiation or their laryngectomy was not done for cancer. In total, there were 48 patients who met inclusion criteria for the study.

Radiation metric data was available for 27 patients; 40/48 patients received radiation treatment at an outside hospital. Total radiation dose varied from 6000 cGy to 7920 cGy (mean 6900 cGy, median 7000 cGy). For 40 patients, it was possible to assess whether there was persistence versus recurrence of tumor. There was persistence of tumor in 13 patients, occurring 1.9 months to 4.8 months after radiation treatment. There was recurrence of tumor in 27 patients, occurring 6.2 months to 24 years after radiation treatment (median 11.4 months). Once recurrence of persistence was diagnosed, salvage surgery was scheduled, with a mean interval from date of diagnosis to surgery of 38 days.

Reconstruction methods were varied: 9 had single-layered primary closure, 14 had double-layered primary closure, 7 had triple-layered primary closure, 3 had pedicled pectoralis myocutaneous inlay flap without free tissue transfer, 9 had pedicled pectoralis myofascial onlay flap without free tissue transfer, and 4 had free tissue transfer. One patient had a temporary esophagostoma and pharyngostoma created in anticipation of future reconstruction, but, for unclear reasons, this patient never had definitive reconstruction. One patient had a stapler assisted closure of the neopharynx. Among patients who had primary closure, the first layer was closed with a running Connell suture. It could not be determined through chart review how the second or third layers were closed if applicable.

Twenty-three of the 48 patients had clinical evidence of a fistula for an overall fistula incidence of 47.9%. Nine patients had a major fistula requiring operative repair for a major fistula rate of 18.8%. Fourteen patients (29.2%) had minor salivary fistulas that resolved without surgical intervention.

We analyzed various preoperative patient and tumor characteristics to determine whether any were associated with fistulas (Table 1). There were no statistically significant associations between overall fistula rate and sex, AJCC tumor stage, or T or N status at the time of initial diagnosis. Age was similar between those who developed fistulas compared to those who did not (64 versus 65 years, *P* = 0.72). There was not a significant difference in fistula rate whether patients received prior radiation at an academic medical center or a community medical center. The use of chemotherapy was not associated with a significant difference in the overall fistula rate. Performing a concurrent neck dissection with the total laryngectomy also was not associated with a significant difference in the overall fistula rate.

Next, comparisons of several closure techniques were made to determine whether differences in fistula rate could be identified (Table 2). There was no significant difference in fistula rate for patients who had a complete pharyngectomy versus those that had either a partial or limited pharyngectomy. The rate of fistula was observed to decrease with increasing number of layers of primary closure, from 66.7% for single-layer closure to 28.6% for triple-layer closures. The fistula rate for pectoralis muscle flap onlay (22.2%) was lower than any of the primary closure techniques. However, none of these differences reached statistical significance.

Major fistulas were analyzed separately for associations with clinicopathologic variables (Table 3). We found that those who developed major fistulas were older (71 versus 63, *P* = 0.03). Sex, AJCC tumor stage, or T or N status at initial diagnosis was not associated with major fistulas. No significant difference in major fistula incidence was detected whether patients were radiated at an academic or community hospital, whether they received chemotherapy, or whether a concurrent neck dissection was performed.

The various neopharyngeal closure techniques were examined for major fistulas (Table 4). Comparing patients who had complete pharyngectomy to those who had a partial or limited pharyngectomy, there was no significant difference in major fistulas. Compared to multilayer primary closures, there was a trend toward a higher major fistula rate with single-layer closures (44.4% versus 12.82%, *P* = 0.09). The rate of major fistula was observed to decrease with increasing number of layers of primary closure, from 44.4% for single-layer closure to 0% for triple-layer closures. No patients (0 of 7) with triple-layer closure had a major fistula, and only 1 of 9 patients (11.1%) with pectoralis muscle flap onlay had a major fistula. However, none of these differences reached statistical significance.

## 4. Discussion

In this study, there were 48 patients who underwent salvage total laryngectomy. Twenty-three (47.9%) patients had a salivary fistula of varying severity. Major salivary fistulas requiring reoperation occurred in 9 patients (18.8%), which is within the broadly documented range of salivary fistula rates (10–50%) reported in the literature [4–19]. It is notable that only a few of these studies delineate how a salivary leak is defined. Fistulas can vary widely in their presentation, from a small leak having minimal impact on postoperative course to large volume salivary drainage leading to prolonged hospital stay and potential catastrophic consequences requiring surgery. The literature pertaining to salivary fistula does not consistently address this variability. For example, Grau et al. defined a fistula as those salivary leaks lasting more than 2 weeks [27]. In our study, we counted any clinically evident fistula regardless of severity or length of time that the leak was present. We also sought to categorize fistulas into minor and major fistulas to determine whether more specific risk factors for salivary leaks could be identified. We did additional analysis on those fistulas requiring operative intervention, which we defined as major fistulas. The way in which the salivary fistula rate is calculated is important since it varies the magnitude of the fistula rate and modifies how we find different risk factors.

Among the preoperative factors studied, our study did not reveal a difference in salivary leak rates with regard to sex, AJCC stage, T or N status at initial diagnosis, or whether radiation treatment was performed at our institution versus a community hospital. Other possible risk factors such as the addition of adjuvant chemotherapy, extent of pharyngeal resection, or concurrent neck dissection were not associated with an increased salivary fistula rate in our study.

Other groups have reported various preoperative factors that are associated with salivary fistula after total laryngectomy. The majority of the literature shows a trend towards higher fistula rates in patients with a history of radiation to the larynx [9–11, 20–22]. The magnitude of the radiation dose seems to be important. In a study by Vendelbo Johansen et al., the fistula rate for salvage total laryngectomy was 25% if patients received 57 Gray (Gy) compared with 92% for those receiving 72 Gy [12]. Other studies have corroborated this finding that higher dose and larger radiation field contribute to fistula formation [27]. In a number of studies, the addition of adjuvant chemotherapy increases by up to twofold the risk of fistula formation when compared to radiation alone [28, 29].

The timing of radiation also seems to affect the likelihood of salivary fistulas. The acute inflammatory effects of radiation can persist for several months after its completion. One study found a significant increase in fistula rate if salvage total laryngectomy was done within 4 months of radiation [30]. Other studies have similarly found a higher wound complication rate for surgeries done soon after radiation [8, 11, 13, 31].

Besides history of radiation or chemoradiotherapy, other factors may put patients at higher risk for salivary fistula. Nonglottic tumors or advanced T3 or T4 tumors tend to have elevated rates of pharyngocutaneous fistula after salvage total laryngectomy [27]. Patients with nutritional deficiencies, hypothyroidism, or hypoalbuminemia are at higher risk as well [4, 19, 32].

The link between postlaryngectomy fistula formation and previous radiation can be explained through radiation therapy's cellular mechanism of action. Radiation induces cell death through DNA damaging mechanisms. Though preferentially affecting rapidly dividing cells such as malignant tumors, radiation also damages normal cells such as connective tissue and muscle. On a microscopic level, radiation leads to progressive fibrosis and obliterative endarteritis of the blood vessels, which in turn inhibits future wound healing. Chemotherapy has been thought to be an effective radiosensitizer, inducing more cellular damage, more fibrosis, and obliteration of the microcirculation [33]. It has been hypothesized that placing nonradiated vascularized tissue into the compromised recipient wound bed can improve wound healing and reduce the incidence of salivary fistulas in those patients who have undergone prior radiation and/or chemotherapy.

We examined whether the neopharyngeal closure technique correlated with the overall (major and minor) fistula rate. We observed a decrease in overall fistulas and major fistulas with increasing the number of primary closure layers. Increasing the number of layers of closure may minimize the risk for the suture line dehiscing. To our knowledge, this is the first study to examine the impact of varying the number of layers of primary closure on fistula incidence for salvage total laryngectomy. However, possibly due to small sample size, none of the differences we observed in fistula rates for the various closure techniques reached statistical significance.

Providing vascularized tissue from outside the previous radiation field as an onlay over the neopharyngeal suture line may facilitate wound healing. Some studies have shown that prophylactically placing a pectoralis myofascial flap over the suture line can reduce the incidence of salivary leak while other studies have failed to find this difference [5–7, 34]. Similar conflicting results have been found with the utilization of free tissue transfer to augment the neopharyngeal suture line [15, 35]. One study showed that placing a pectoralis flap in an inlay fashion reduced the salivary leak rate when compared to an onlay fashion, citing that skin holds sutures better than fascia or muscle [6, 24]. All these studies suffer from low statistical power and lack a standardized method of defining salivary leaks. Placing vascularized tissue in the wound bed may not reduce the overall incidence of salivary fistula, but it may mitigate the severity of the leak. Perhaps prophylactic placement of vascularized tissue converts cases that would have resulted in a severe fistula into a mild fistula that can be managed conservatively [35]. In our study, the use of a pedicled pectoralis muscle onlay flap was noted to reduce the overall and major fistula rates compared to single-layer closures; however the difference was not significant possibly due to small sample size. Nonetheless, it seems likely that certain subgroups of patients more prone to poor wound healing would benefit from vascularized tissue overlying the neopharyngeal suture line. Further studies are needed to explore and define appropriate recommendations for closure techniques in salvage total laryngectomy.

It is also interesting to speculate whether a multilayer closure for total laryngectomy in patients without a history of prior radiation treatment has an impact on the occurrence of salivary leaks. Salivary fistulas are much less common in this population. A future study could help determine whether there is any added benefit to performing more than a single-layer closure for previously untreated patients undergoing total laryngectomy.

This study has several limitations. This study is retrospective with inherent selection biases. The utilization of various closure techniques may have been predicated on certain preoperative or intraoperative findings that raised the surgeon's fear of having a fistula. Our study had a small sample size and was thus underpowered to detect statistical differences that may truly exist. We observed a trend with greater number of layers of neopharyngeal closure techniques or onlay pectoralis flaps and decreased rates of pharyngocutaneous fistula, findings that may have been statistically significant with a larger sample size. These limitations are not unique to this study, and the majority of single-institution reports on this topic are afflicted with these same drawbacks.

Although there is a paucity of data from the literature to guide the surgeon as to how to prevent pharyngocutaneous fistulas after salvage total laryngectomy, we believe that the optimal management of these challenging cases should begin with identifying those patients at highest risk for developing fistulas. The literature suggests that this high risk group includes patients who have been treated previously with chemoradiation or those with poor nutritional status. In these patients, the surgeon should carefully select the method of neopharyngeal reconstruction to decrease the risk of fistula. Our study suggests that it may be beneficial to increase the number of pharyngeal closure layers or to use a pedicled pectoralis muscle onlay flap.

## 5. Conclusions

In this study, salivary fistulas were a common complication after salvage total laryngectomy, occurring with varying severity in 47.9% of cases. Reoperation due to salivary fistulas was performed for nearly 1 in 5 salvage total laryngectomies. In contrast to previous studies, we did not find any clinicopathologic variables associated with fistulas, which may be related to the small sample size. The overall fistula and major fistula incidence was decreased with increasing the number of layers of primary closure and with pectoralis muscle onlay flaps. These observations warrant further study to establish their significance.

## Figures and Tables

**Table 1:** Preoperative clinicopathologic data for patients who had any type of salivary fistula (major or minor).

Major/minor fistula pooled comparisons	Category	Fistula rate	*P* value
Sex	Males	44.7% (17/38)	0.61
	Females	60% (6/10)	

Stage at Dx	1	46.7% (7/15)	0.77
	2	58.3% (7/12)	
	3	40% (2/5)	
	4	55.6% (5/9)	

T stage at Dx	T1	46.7% (7/15)	0.78
	T2	52.9% (9/17)	
	T3	44.4% (4/9)	
	T4	66.7% (2/3)	

N stage at Dx	N0	53.1% (17/32)	0.75
	N1	0.00% (0/2)	
	N2B	66.7% (2/3)	
	N2C	50.0% (3/6)	

Tx at AC	No	42.5% (17/40)	0.13
	Yes	75.0% (6/8)	

Chemotherapy/ radiation	XRT alone	56.00% (14/25)	0.39
	ChemoXRT	40.90% (9/22)	

Type of surgery	TL alone	57.10% (4/7)	0.70
	TL and neck dissections	46.30% (19/41)	

Dx: diagnosis; Tx: treatment; AC: academic center; XRT: radiation; ChemoXRT: chemoradiation; TL: total laryngectomy.

**Table 2:** Comparisons between types of neopharyngeal closure techniques and their associated overall fistula rate (major and minor).

Minor/major fistula pooled comparisons	Fistula rate	*P* value
Complete pharyngectomy vs. partial	50.0% (1/2) vs. 45.5% (5/11)	0.51
Single-layer closure vs. all others	66.7% (6/9) vs. 43.6% (17/39)	0.38
Single- vs. double-layer closure	66.7% (6/9) vs. 57.1% (8/14)	0.98
Single- vs. triple-layer closure	66.7% (6/9) vs. 28.6% (2/7)	0.31
Single-layer closure vs. pec. flap overlay	66.7% (6/9) vs. 22.2% (2/9)	0.15
Pec. flap overlay vs. all others	22.2% (2/9) vs. 53.8% (21/39)	0.18

vs.: versus; pec.: pectoralis.

**Table 3:** Preoperative clinicopathologic data for patients who had major fistulas.

Major fistula only comparisons	Category	Fistula rate	*P* value
Sex	Males	21.10% (8/38)	0.73
	Females	10% (1/10)	

Stage at Dx	1	13.30% (2/15)	0.43
	2	33.30% (4/12)	
	3	0% (0/5)	
	4	33.30% (3/9)	

T stage at Dx	T1	13.30% (2/15)	0.68
	T2	29.40% (5/17)	
	T3	11.10% (1/9)	
	T4	33.30% (1/3)	

N stage at Dx	N0	18.80% (6/32)	0.38
	N1	0% (0/2)	
	N2B	0% (0/3)	
	N2C	50.0% (3/6)	

Tx at AC	No	15.0% (6/40)	0.16
	Yes	37.50% (3/8)	

Chemotherapy/ radiation	XRT alone	24.0% (6/25)	0.47
	ChemoXRT	13.60% (3/22)	

Type of surgery	TL alone	0% (0/7)	0.32
	TL and neck dissections	22.0% (9/41)	

Dx: diagnosis; Tx: treatment; AC: academic center; XRT: radiation; ChemoXRT: chemoradiation; TL: total laryngectomy.

**Table 4:** Comparisons between types of neopharyngeal closure techniques and their associated major fistula rate.

Major fistula only comparisons	Fistula rate	*P* value
Complete pharyngectomy vs. partial	0% (0/2) vs. 27.27% (3/11)	0.40
Single-layer closure vs. all others	44.4% (4/9) vs. 12.82% (5/39)	0.09
Single- vs. double-layer closure	44.4% (4/9) vs. 14.3% (2/14)	0.26
Single- vs. triple-layer closure	44.4% (4/9) vs. 0.0% (0/7)	0.15
Single-layer closure vs. pec. flap overlay	44.4% (4/9) vs. 11.1% (1/9)	0.29
Pec. flap overlay vs. all others	11.1% (1/9) vs. 20.5% (8/39)	0.86

vs.: versus; pec.: pectoralis.
